# Preparation and characterization of zein–lecithin–total flavonoids from *Smilax glabra* complex nanoparticles and the study of their antioxidant activity on HepG2 cells

**DOI:** 10.1016/j.fochx.2023.100579

**Published:** 2023-01-18

**Authors:** Jing Li, Yingxiu Zhang, Wenfang Jin, Yue Wang, Li Yang, Zhifeng Zhang, Zhigang Yan

**Affiliations:** a School of Pharmacy, Southwest Minzu University, Chengdu 610225, PR China; b National Engineering Institute for the Research and Development of Endangered Medicinal Resources in Southwest China, Guangxi Botanical Garden of Medicinal Plants, Nanning 530023, China; c Tibetan Plateau Ethnic Medicinal Resources Protection and Utilization Key Laboratory of National Ethnic Affairs Commission of the People's Republic of China, Southwest Minzu University, Chengdu 610225, PR China

**Keywords:** Total flavonoids, *Smilax glabra*, Self-assembled nanoparticles, Stability, Antioxidant activity

## Abstract

Total flavonoids from *Smilax glabra* (TFSG) exhibit several biological activities; however, their poor stability limits their application. In this work, zein–lecithin–TFSG complex nanoparticles (Z–L–TFSG NPs) were prepared using the anti-solvent coprecipitation technique. The prepared Z–L–TFSG NPs were spherical with an encapsulation efficiency of 98.0%. Differential scanning calorimetry, Fourier transform infrared spectroscopy, and morphology tests revealed that the TFSG were successfully encapsulated by Z–L NPs. Z–L–TFSG NPs showed superior stability and better controlled release characteristics in simulated gastrointestinal digestion. The encapsulation of TFSG by Z–L NPs could improve their antioxidant capacity *in vitro*. Moreover, Z–L–TFSG NPs could enhance the protective effects of TFSG against H_2_O_2_-induced oxidative damage to HepG2 cells. The results indicated that the Z–L self-assembled NPs could serve as a promising drug delivery system through the integrated encapsulation of multiple flavonoids.

## Highlights

• New Z-L-TFSG complex nanoparticles were prepared by the anti-solvent coprecipitation method.
• The particles exhibited nano-scale size and excellent storage stability.
• The nanoparticles had sustained release in a simulated gastrointestinal tract.
• Antioxidant performance of Z-L-TFSG NPs *in vitro* was improved after encapsulation.

## Introduction

*Smilax glabra* Roxb. (SG), called Tufuling in Chinese, is a rhizome of the Liliaceae plant and belongs to the Smilacaceae family. It was used as an edible plant and herbal medicine for hundreds of years in China and other Asian countries (Hua et al., 2018). SG was also used as a food ingredient that was added to teas, functional foods, and sanitarian soups. As a functional food with great nutritional and medicinal value, SG is one of the main components of turtle jelly (Guilinggao) prepared in the southern regions of China. SG was widely used in traditional Chinese medicine in the treatment of nephritis, syphilis, heavy metal poisoning, hypertonia, and other diseases. In fact, SG was shipped to Europe for syphilis treatment in the 16th century (Zhao et al., 2020a). In Hong Kong, Macau, and the Guangdong and Guangxi provinces of China, turtle jelly is widely used as a medicinal diet to nourish the body by reducing body heat and removing toxins in the blood. Some recent studies have suggested that the chemical constituents of SG exhibit antioxidative (Shi, et al., 2020), antibacterial, anti-inflammatory, anti-gout, antiviral, and hypouricemic properties, and also provide cardiovascular protection and hepatoprotection (Feng et al., 2020, Huang et al., 2019).

Flavonoids are one of the main ingredients of SG (Zhao et al., 2020a). More than 20 different flavonoids and flavonoid glycosides have been separated and identified from SG in previous studies, such as astilbin, isoastilbin, neoastilbin, neoisoastilbin, engeletin, isoengelitin, taxifolin, and quercetin (Shu et al., 2018, Xu et al., 2013). Astilbin is usually considered the main bioactive compound in SG, and has three stereoisomers (neoisoastilbin, isoastilbin, and neoastilbin) that exist simultaneously in SG (Zheng, Zhang, & Zhang, 2018). Free radical-mediated oxidative stress could be involved in the pathogenesis of many diseases, such as inflammation, cancer, and neurodegenerative disorders (McCord, 2000). It was reported that the flavonoids of SG, especially astilbin and its stereoisomers, exhibit significant antioxidant activity (Shu et al., 2018). However, astilbin and its stereoisomers are highly unstable, and, therefore, prone to mutual transformation due to the similarity in their structures, which greatly limits their applications in medicine and food (Zhang, Fu, Huang, Shangguan, & Guo, 2013). Hence, improving the stability of the flavonoids of SG is critical for their practical application. Some studies have shown that the molecular structure of flavonoids can be changed by chemical reaction to improve their storage stability; however, this method was considered unsuitable since the safety of the modified products could not be guaranteed (Hussain, Hadi, & Akbar, 2019). To overcome this shortcoming, active substances are usually embedded in various nanomaterials (such as nanoparticles, microcapsules, nanolotions, and hydrogels) in which nanoparticles (NPs) are widely used (Hussain et al., 2019). Moreover, since Chinese medicine emphasizes multicomponent and multitarget synergy, these flavonoids are often used as a whole to exert a curative effect. Therefore, we need to find a new delivery system for the integrated encapsulation of multiple flavonoids in SG.

Zein is the main storage protein in corn and contains >50 % of hydrophobic amino acids, which make it insoluble in water but soluble in aqueous ethanol. It was observed that zein loses its solubility and can self-assemble into NPs with a decrease in the concentration of ethanol (Luo & Wang, 2014). Lecithin is believed to be a biocompatible and safe excipient and has been used in the pharmaceutical, food, and cosmetic industries. Lecithin is an amphiphilic molecule composed of a hydrophilic head (phosphatidyl substituent) and a hydrophobic tail (fatty acid chain) that can interact with zein and form stable composite colloidal NPs in aqueous ethanol (Dai, Sun, Wang, & Gao, 2016). A previous study reported that epigallocatechin gallate loaded with zein and lecithin NPs showed excellent stability and great sustained-release performance (Xie et al., 2021). Astilbin was encapsulateed as a model flavonoid by preparing the core–shell zein NPs with lecithin; the encapsulation improved the stability of astilbin and also increased its bioavailability both *in vitro* and *in vivo* (Ruan et al., 2021). However, currently, there are no published results of the studies conducted on the self-assembled NPs of the total flavonoids from *S. glabra* (TFSG).

**Fig. 1:**
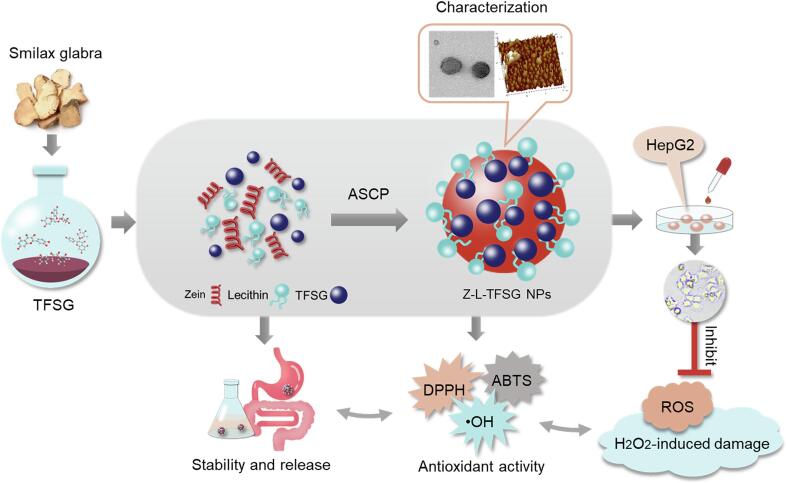
An illustration of the possible structures of Z–L–TFSG NPs and their antioxidant activities. ASCP, anti-solvent coprecipitation; TFSG, total flavonoids from *S. glabra*; Z–L–TFSG NPs; zein–lecithin–TFSG complex nanoparticles.

In the present study (Fig. 1), TFSG was extracted and purified, and the zein–lecithin–TFSG complex nanoparticles (Z–L–TFSG NPs) were prepared using the anti-solvent coprecipitation (ASCP) technique. The zeta potential, particle size, morphology, and structure of the prepared NPs were obtained. The characteristics and antioxidant activity of the composite NPs were evaluated. Furthermore, the antioxidant activity of Z–L–TFSG NPs on the HepG2 cell model was evaluated. We believe that our findings will provide some basis for developing a new kind of safe and effective delivery system for the integrated encapsulation of multiple flavonoids.

## Materials and methods

### Materials

Lecithin (purity > 98 %) and zein (purity > 98 %) were purchased from Macklin (Shanghai, China). Astilbin (purity > 98 %) was purchased from Kangbang (Chengdu, China). 30 % Hydrogen peroxide was purchased from Jingshan (Chengdu, China). Salicylic acid, KCl, NaCl, acetic acid, ethanol, ascorbic acid, and macroporous resin D101 were purchased from Kelong (Chengdu, China). 2,2-Diphenyl-1-picrylhydrazyl (DPPH) was purchased from Basifu (Hefei, China). 2,2′-Azino-bis (3-ethylbenzothiazoline-6-sulfonic acid) (ABTS) was purchased from Solarbio (Beijing, China). Penicillin-streptomycin was purchased from Hyclone (Logan, UT, USA). Dulbecco’s modified essential medium (DMEM, high glucose), phosphate buffered saline (PBS), and trypsin digest were purchased from Servicebio (Wuhan, China). HepG2 cells and fetal bovine serum were provided by Procell (Wuhan, China). The reactive oxygen species detection assay kit was purchased from Biyuntian (Shanghai, China). Dimethyl sulfoxide (DMSO‑*d*_6_, 99.9 %) were purchased from Siyituopu (Wuhan, China). Ultrapure water was obtained using the Milli-Q system (Milli-pore, USA). All other reagents used were of analytical grade.

### Preparation of total flavonoids of Smilax glabra

The *S. glabra* samples were collected from Guangxi province and verified by Prof. Zhifeng Zhang (College of Pharmacy, Southwest Minzu University, Chengdu, China). The dried sample (5 kg, cut into thin slices) was extracted with 70 % ethanol–water (v/v) three times (100 L, 60 °C for 2 h). The extracts were filtered and collected, and the combined filtrate was evaporated in a rotary evaporator at 50 °C to obtain the ethanolic extract of *S. glabra* (EESG). The extract was dissolved in distilled water and the pH was adjusted to 5.0 using phosphoric acid. EESG was purified using the D101 macroporous resin. The combined filtrate was concentrated to extract under reduced pressure and freeze-dried to obtain total flavonoids of Smilax glabra (TFSG) powder.

The sodium nitrite–aluminum nitrate colorimetric method was used to determine the total flavonoids in the TFSG powder. In a 10 mL test tube, 1 mL of the sample, 1 mL NaNO_2_ (5 %), and 1 mL Al_2_(NO_3_)_3_ (10 %) were mixed; 4 mL NaOH (4 %) was added to the solution and mixed well. The absorbance was measured at 510 nm against the reagent blank. Rutin standard solution (0–100 mg/L) was used to make the standard curve for total flavonoids. The flavonoids were expressed as milligrams of rutin equivalents per gram of the dried fraction. Astilbin and its stereoisomers (isoastilbin, neoisoastilbin, and neoastilbin) in TFSG were detected using ultra-high performance liquid chromatography (UPLC, 1290, Agilent, USA). The chromatographic separation was performed using a C18 column (2.1 × 100 mm, 1.7 um column). The mobile phase consisted of (A) acetonitrile and (B) 0.1 % formic acid in water. The optimized UPLC elution conditions were as follows: 10–15 % A (0–2 min), 15–15 % A (2–5 min), 15–20 % A (5–7 min), 20–20 % A (7–10 min), 20–25 % A (10–11 min), 25–25 % A (11–12 min), 25–80 % A (12–15 min), and 80–10 % A (15–18 min). The column was maintained at 30 °C, the flow rate was set at 0.3 mL/min, and the UV detection was set at 306 nm. The injection volume was 0.5 μL (Zhao et al., 2020a).

### Preparation of Zein-lecithin-TFSG complex nanoparticles

Zein (1.0 g) was added to 500 mL of 75 % methanol–water (v/v) with continuous stirring using a magnetic stirrer for 2 h (700 rpm) (Zhao et al., 2020b). Then different amounts of lecithin were added to the prepared zein solution and the resulting solutions were stirred using a magnetic stirrer for 3 h to reach zein:lecithin mass ratios of 1:0 (S1), 2:1 (S2), 3:2 (S3), 1:1 (S4), 2:3 (S5), 1:2 (S6), and 0:1 (S7), respectively. Following this, 1.0 mL TFSG–water solution (100 mg/mL) was added to the prepared zein–lecithin (Z–L) solutions, stirred continuously for 1 h, and then concentrated to 20 mL to obtain the Z–L–TFSG NPs. The obtained 20 mL NPs were slowly injected into 60 mL deionized water with constant stirring for 0.5 h (700 rpm). The remaining ethanol was evaporated using a rotary evaporator at 50 °C to form composite nanoparticle dispersions, and the pH value of the dispersions was adjusted to 4.0 by adding NaOH (0.1 mol/mL) or HCl (0.1 mol/mL). The dispersions were subjected to centrifugation at 2000 rpm for 20 min to separate free TFSG and large particles (Xie et al., 2021). The composite nanoparticle dispersions were kept at 4 °C, and some of the dispersions were freeze-dried to obtain the corresponding powders.

### Determination of zeta potential, particle size, and polydispersity index

One milliliter of Z–L–TFSG NP dispersions from each group (S1–S7) was added to 20 mL of distilled water to obtain the corresponding dilute solutions. The zeta potential, particle size, and PDI of the diluted solutions from each group were determined using a combined method comprising dynamic light scattering (DLS) and particle electrophoresis (Zetasizer Nano-ZS, Malvern, UK). The zeta potential and particle size of the 1.5 mL samples were repeatedly measured three times. All measurements were carried out at 25 °C.

### Storage stability

The Z–L–TFSG NP dispersions from each group were stored in a refrigerator at 4 °C for 14 days. The particle size and PDI of the dispersions in each group were observed on the 14th day of the experiment.

### Encapsulation efficiency and loading capacity

One milliliter of nanoparticle dispersions from each group was diluted with 7 mL 75 % methanol–water (v/v) using ultrasonication, and the resultant solution was centrifuged at 20 °C at a high-speed centrifuge for 10 min (5000 rpm). The absorbance-unincorporated TFSG in each sample was detected using the sodium nitrite-aluminum nitrate colorimetric method, and astilbin and its stereoisomers were detected using UPLC. The Encapsulation efficiency (EE) and loading capacity (LC) values were obtained from Eqs. (1) and (2), respectively (Radwan, Elmaadawy, Yousry, Elmeshad, & Shoukri, 2020):

(1) $$EE\;\left(\%\right)=\frac{W_{\mathrm{total}}\;-\;W_{\mathrm{free}}}{W_{\mathrm{total}}}\times 100\%$$

(2) $$LC\;\left(\%\right)=\frac{W_{\mathrm{total}}\;-\;W_{\mathrm{free}}}{W_{Z-L}\;}\times 100\%$$

where *W*_total_ is the total amount of the required drug in the solution, W_free_ is the amount of free drug in the supernatant, and WZ–L is the total amount of Z–L.

### Fourier transform infrared spectroscopy

All Fourier transform infrared (FTIR) spectra were recorded on an FTIR spectrometer (Thermo Fisher, USA) using the attenuated total reflection method. The FTIR samples were prepared by mixing the optimized complex nanoparticle samples (2 mg) with spectroscopic grade KBr (198 mg), and were compressed to the sheet. The spectrum of KBr powder without drugs was determined as the blank baseline. The measurement was performed in the range of 4000–400 cm^−1^ with a spectral resolution of 4 cm^−1^, with 64 scans being collected for one spectrum.

### Nuclear magnetic resonance spectroscopy measurement

All ^1^H NMR spectra were recorded on a Nuclear Magnetic Resonance (NMR) Spectrometer (DD2 400-MR, Agilent, USA), operating at 400.13 MHz with pulsed field gradient capabilities for hydrogen. The NMR samples were firstly evaporated under a stream of nitrogen and then in vacuum (overnight), then dissolved in deuterated DMSO. ^1^H NMR spectra were acquired at an acquisition time of 3.2 s and a recycle delay of 1 s acquiring 64 K data points. ^1^H NMR spectra of TFSG and Z–L–TFSG NPs were collected.

### Differential scanning calorimetry

The physical state of the optimized TFSG, Z–L, and Z–L–TFSG NPs was determined using differential scanning calorimetry (DSC, Q2000, Thermo Fisher, USA). Accurately weighed, freeze-dried samples of 3 mg were placed in an aluminum pan, and an empty, sealed aluminum pan was used as the reference. The samples were heated from –20 °C to 200 °C at a heating rate of 10 °C/min under nitrogen gas flow. DSC thermograms of the physical mixtures of TFSG, Z–L, and Z–L–TFSG NPs were collected.

### Transmission electron microscopy

The shape and the surface morphology of the optimized Z–L and Z–L–TFSG NPs were analyzed by Transmission electron microscopy (TEM, H77800, Hitachi, Tokyo, Japan). The samples were first dropped on a copper 400-mesh coated with carbon film; the mesh was then stained with 2 % phosphotungstic acid for 3 min. The freshly stained samples were then observed and photographed.

### Atomic force microscopy

The morphology of Z–L and Z–L–TFSG NPs was analyzed using atomic force microscopy (AFM, Dimension Icon, Bruker, Germany). The analysis was performed in the air under contact mode using a Sharp Nitride Lever probe with a spring constant of 0.04 N/m. The samples were sonicated, deposited on freshly cleaved mica substrates, and dried with the help of compressed air.

### Determinants of antioxidant activity

#### DPPH radical scavenging activity

TFSG and powders of Z–L–TFSG NPs were dissolved in distilled water and 75 % methanol–water (v/v) to obtain the corresponding TFSG (0.3 mg/mL) and Z–L–TFSG NP (0.3 mg/mL, calculated as TFSG) solutions. Four milliliters of the samples (TFSG or Z–L–TFSG NPs) were added to 4 mL DPPH–ethanol solution (0.1 mmol/L) and mixed thoroughly. The solutions obtained were incubated in dark for 30 min. They were then analyzed at different intervals of time (0, 30, 60, 120, and 240 min). The absorbance values of the sample solutions were determined at 519 nm on an ultraviolet–visible (UV) spectrophotometer (A_sample_). The samples mixed with distilled water were used as the blank (A_blank_). The mixed solution of DPPH and distilled water was used as the control (A_control_). The scavenging rate (Donsì et al., 2017, Song et al., 2022, Kao et al., 2019) was determined using the following formula:

(3) $$DPPH\;scavenging\;ability\;\left(100\%\right)=\left(1-\frac{A_{sample}-A_{blank}}{A_{control}}\right)$$

#### ABTS radical scavenging activity

ABTS free radicals were obtained by mixing potassium persulfate (2.6 mmol/L) with an equal volume of ABTS (7.4 mmol/L) and placing the solution in darkness for 12 h. The working solution of ABTS was prepared by diluting the obtained solution with phosphate-buffered saline buffer (PBS, pH7.4) to an absorbance value of 0.70 (734 nm) and placed at 4 °C. The TFSG and Z–L–TFSG NP solutions (as described in Section 2.12.1) were added to 50 mL of distilled water to make sample solutions. Two milliliters of the ABTS working solution was mixed with 3 mL of the sample solutions and reacted for 10 min (A_sample_). The obtained solutions were analyzed at different intervals of time (0, 30, 60, 120, and 240 min). The ABTS–water solution was used as a blank (A_blank_). The absorbance values of the above solutions were determined at 734 nm using UV spectroscopy (Saeed et al., 2012, Yi et al., 2022) and their scavenging activities were obtained using the following formula:

(4) $$ABTS\;scavenging\;ability\;\left(100\%\right)=\frac{{A_{blank}-A}_{sample}}{A_{blank}}$$

#### Hydroxyl radical scavenging activity

Hydroxyl radicals (·OH) were generated by carrying out a classical Fenton reaction in a freshly prepared reaction mixture containing H_2_O_2_ and FeSO_4_. Salicylic acid was used to react with the ·OHs, which yielded 2,3-dihydroxybenzoic acid with a typical UV absorption at 510 nm. The absorption of the sample solutions was compared with that of the blank solution to determine the hydroxyl radical scavenging activity. To make the sample solutions, first, 1 mL FeSO_4_ (9 mmol/L) and salicylic acid–ethanol solution (9 mmol/L) were taken in the colorimetric tubes; TFSG and Z–L–TFSG NPs solutions were then added to the colorimetric tubes (as described in Section 2.12.1); finally, 1 mL H_2_O_2_ (8.8 mmol/L) was added to the colorimetric tubes and the solutions were diluted to 15 mL by adding distilled water. The tubes were heated in a water bath at 40 °C for 30 min; the solutions were analyzed at different intervals of time (0, 30, 60, 120, and 240 min) (Saeed et al., 2012). The absorbance values of the samples were determined at 510 nm by UV spectroscopy (A_sample_) and the absorbance of the solution without the samples was used as the blank (A_blank_). The free radical scavenging activity (%) was obtained using the following equation:

(5) $$Hydroxyl\;radical\;scavenging\;ability\;\left(100\%\right)=\left(1-\frac{A_{sample}}{A_{blank}}\right)$$

### In vitro controlled release study

The controlled release of free TFSG, TFSG in Z-TFSG NPs, and TFSG in Z–L–TFSG NPs was carried out under simulated gastrointestinal conditions (Wang et al., 2021). Three milliliters of the prepared samples were sealed in a dialysis bag (3500 Da molecular cut-off) and then incubated in a flask with 150 mL simulated gastric fluid (SGF, pepsin aqueous solution, 15 mg/mL) with gentle shaking for 2 h (37 °C). The dialysis bag with the sample was transferred to another flask containing 150 mL of the simulated intestinal fluid (SIF; pancreatin aqueous solution, 30 mg/mL) and incubated for 9 h (37 °C). The TFSG content in a 1 mL release medium was determined using the sodium nitrite–aluminum nitrate colorimetric method (as described in Section 2.2). Equal volumes of fresh simulated fluids were added to the flask to maintain a constant volume (Li et al., 2019). The drug release rate (%) was calculated using the following equation:

(6) $$Drug\;release\;\left(100\%\right)=\frac{C\times V}{M}$$

where C is the concentration (mg/mL) of the drug in the solution, V is the volume (mL) of the solution, and M is the amount of drug (mg) in the complex NPs.

### Cytotoxicity assay

The cytotoxicity of TFSG and Z–L–TFSG NPs was determined by the cell counting kit-8 (CCK-8) assay using HepG2 cells. The HepG2 cells were cultured in the DMEM medium (high glucose) with 1 % penicillin–streptomycin and 10 % fetal bovine serum. After reaching 80 % confluence, the cells were digested with 0.25 % trypsin to produce a single cell suspension; 100 μL of the suspension (1 × 10^4^ cells/mL) was seeded into a 96-well plate and incubated at 37 °C with 5 % CO_2_, 95 % air, and 100 % relative humidity, to allow for cell attachment. The cells were then treated with serial concentrations of TFSG (0, 0.5, 1, 1.5, 2, 5, and 10 mg/mL) or Z–L–TFSG NPs (0, 0.5, 1, 1.5, 2, 5, and 10 mg/mL; calculated as TFSG) for 24 h. Following this, 10 μL of the CCK-8 solution was added to each well, and the cells were incubated for an additional 4 h in an incubator. The optical density was recorded at 450 nm using a microplate reader (MQX200, BIO-TEK, USA). Cell viability was obtained using the following equation:

(7) $$Cell\;viability\left(100\%\right)=\frac{A_{sample}-A_{blank}}{A_{control}-A_{blank}}$$

where A_control_ is the absorbance of the negative control, A_sample_ is the absorbance of the HepG2 cells treated with TFSG or Z–L–TFSG NPs, and A_blank_ is the absorbance of the blank sample.

### Protective effects of Z–L–TFSG NPs against H_2_O_2_-induced oxidative damage

***Selection of H2O2 concentration in the oxidative damage model***

The HepG2 cells (1 × 10^4^ cells/mL) were incubated in a 96-well plate at 37 °C for 24 h. The cells were then treated with H_2_O_2_ (0, 100, 200, 300, 400, 500, 800, and 1000 μmol/L) for 24 h. Following this, 10 μL CCK-8 solution was added to each well, and the cells were incubated for another 4 h. Cell viability was evaluated using the CCK-8 assay.

#### Determination of H_2_O_2_-induced oxidative damage in HepG2 cells

The HepG2 cells (1 × 10^4^ cells/mL) were cultured in a 96-well plate at 37 °C for 24 h. The cells were then treated with the blank group, control group (400 μmol/L H_2_O_2_), TFSG group (0.5, 1, 1.5, 2, 5, and 10 mg/mL), and the Z–L–TFSG NPs (0.5, 1, 1.5, 2, 5, and 10 mg/mL; calculated as TFSG) for 1 h. Then, H_2_O_2_ was added until the final concentration was 400 μmol/L (total volume of 200 μL). After incubation for 24 h, 10 μL CCK-8 solution was added to each well, and the cells were incubated for another 4 h. Cell viability was evaluated using the CCK-8 assay.

### *Determination of H*_*2*_*O*_*2*_*-induced reactive oxygen in HepG2 cells*

The HepG2 cells (1 × 10^4^ cells/mL) were cultured in a 96-well plate at 37 °C for 24 h. The cells were then treated with the blank group, control group (400 μmol/L H_2_O_2_), TFSG group (0.5, 1, 1.5, 2, 5, and 10 mg/mL), and the Z–L–TFSG NPs (0.5, 1, 1.5, 2, 5, and 10 mg/mL; calculated as TFSG) for 1 h. Then, H_2_O_2_ was added to make the final concentration 400 μmol/L (total volume of 200 μL). The culture medium was discarded after incubation for 24 h, and the cells were washed three times with 200 μL DMEM. Following this, each well was filled with a 150 μL DCFH-DA fluorescent probe (μmol/L). After incubating at 37 °C in the dark for 20 min, the cells were washed three times using cool PBS. After the PBS was absorbed in the wells, reactive oxygen production was measured using a fluorescence inverted microscope (M152-N, MSHOT, China).

### Statistical analysis

Data were reported as mean ± standard deviation, and statistical analyses were performed by one-way analysis of variance (ANOVA) using the GraphPad Prism 8 software. Differences were considered to be significant when *P* < 0.05.

## Results and discussion

### Extraction and purification of total flavonoids from S. Glabra

The content of total flavonoids was 17.1 % when extracted using ethanol. After purification using the D101 macroporous resin, the content of total flavonoids increased to 60.59 %. The results showed that the D101 macroporous resin purified TFSG efficiently. In our research, the maximum amount of total flavonoids was obtained when the pH of the sample solution was 5, indicating that TFSG, which is a weak acidic substance, exhibits a good adsorption effect in an acidic environment.

### Particle size, zeta potential, and PDI

The effects of zein:lecithin mass ratios on the zeta potential, particle size, and PDI of the NPs are presented in Table 1. The average particle size of the Z–TFSG and l–TFSG NPs was around 160 and 118 nm, respectively. The particle size of Z–L–TFSG NPs varied with the zein: lecithin mass ratios. With the increase in the amount of lecithin, the particle size of Z–L–TFSG NPs increased at first and then decreased. When the mass ratios of zein:lecithin were 2:1, 3:2, and 1:1, the particle sizes of Z–L–TFSG NPs were larger (216, 272, and 278 nm, respectively) than that of the Z–TFSG NPs (160 nm). It was reported that the C16 alkyl chain of sodium stearate may be mounted onto the hydrophobic region of zein to obtain a zein sodium stearate complex particle with partially unfolded hydrophobic microregions, and an increase in anisotropy was accompanied by the partial unfolding of zein, which could contribute to the large particle size (Gao et al., 2014). Further increasing the lecithin mass ratio led to a significant reduction in the particle size from 278 to 132 and 139 nm for the zein: lecithin mass ratios of 2:3 and 1:2 Z–L–TFSG NPs, respectively. One explanation for this is that lecithin and zein could form a compact structure at a relatively high mass ratio of lecithin, which could decrease the size of composite NPs. Our results were consistent with those of a previous study which reported that the zein: lecithin mass ratios exhibit the same effects on the particle size when curcumin was entrapped in the Z–L NPs (Dai et al., 2017). As shown in Table 1, PDI exhibited a similar trend as particle size. The PDI of the NPs was < 0.2 when the mass ratios of zein: lecithin were between 2:3 and 1:2, which indicated that the particle sizes of the NPs were uniform.

**Table 1:** Particle size, polydispersity index (PDI), zeta-potential, encapsulation efficiency (EE) and loading loading capacity (LC) of Zein-lecithin and Zein-lecithin-TFSG systems.

Samples	Particle size (nm)	PDI	Zeta-potential (mV)	EE (%)	LC (%)
S1(Z/L_1:0_)	160.37 ± 1.31	0.54 ± 0.023	−35.90 ± 0.52	72.08 ± 5.00	7.21 ± 0.80
S2(Z/L_2:1_)	216.60 ± 0.86	0.31 ± 0.022	−38.30 ± 0.70	73.50 ± 3.50	2.48 ± 0.50
S3(Z/L_3:2_)	272.47 ± 1.73	0.29 ± 0.014	−34.17 ± 0.71	76.24 ± 7.01	3.02 ± 1.00
S4(Z/L_1:1_)	278.27 ± 7.29	0.26 ± 0.007	−37.17 ± 1.05	95.12 ± 2.01	4.72 ± 0.50
S5(Z/L_2:3_)	131.63 ± 1.88	0.18 ± 0.010	−44.13 ± 0.90	98.00 ± 1.00	4.00 ± 0.26
S6(Z/L_1:2_)	139.23 ± 0.61	0.19 ± 0.004	−46.83 ± 0.33	97.64 ± 1.52	3.97 ± 1.00
S7(Z/L_0:1_)	118.10 ± 0.99	0.67 ± 0.026	−44.20 ± 0.49	96.89 ± 2.59	9.75 ± 0.75

As summarized in Table 1, the zeta potentials of the Z–TFSG and l–TFSG NPs were –35.9 and –44.2 mV, respectively. When the zein: lecithin mass ratio varied from 2:1 to 1:2, the zeta potential of the Z–L–TFSG NPs changed from –34.2 to –46.8 mV. The zeta potential reflects the stability of the NP emulsions. If the nanoparticle emulsions have a high absolute value of zeta potential (positive or negative), the emulsion is more stable and aggregation of the NPs is more difficult (Siddiqui, Alayoubi, El-Malah, & Nazzal, 2014). Generally, NPs with a zeta potential > 30 mV (absolute value) will have good colloidal stability due to sufficient electrostatic repulsion; when the mass ratio of zein: lecithin changed from 1:1 to 2:3, the zeta potential of Z–L–TFSG NPs changed from –37.1 to –44.1 mV. Further increasing the lecithin level rarely showed any influence on the zeta potential of the NP emulsions.

Based on the above results, the Z–L–TFSG NPs with higher concentrations of lecithin (zein: lecithin mass ratios of 2:3 or 1:2) were associated with lower particle size, PDI, and higher zeta potential, which contributed to the improved stability of the NP emulsion. The improved stability was because using lecithin as a surfactant improved the emulsifying effect of the NP emulsion. When the zeta potential (absolute value) of the NPs increases, the repulsive force among the particles increases, and the particle size decreases; consequently, the NP emulsions become more stable and the aggregation of the NPs becomes more difficult.

### Encapsulation efficiency and loading capacity

**Fig. 2:**
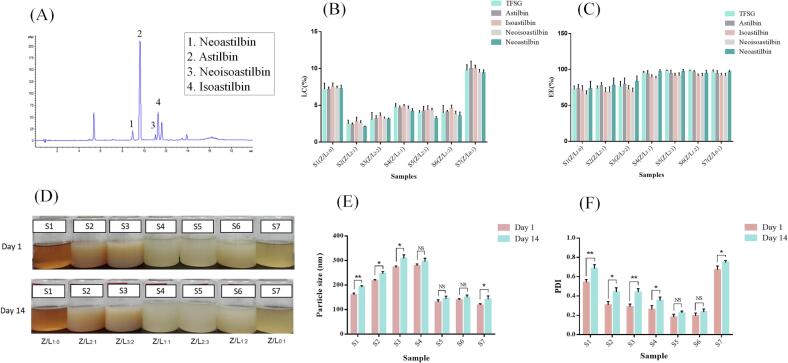
Encapsulation efficiency, loading capacity and storage stability of Z-L-TFSG NPs. UPLC chromatogram of astilbin and its stereoisomers in TFSG (A), encapsulation efficiency (B) and loading capacity (C) of TFSG, astilbin and its stereoisomers in Z-L-TFSG systems. The change of precipitation(D), PDI (E), and particle size (F) of complex nanoparticles in each groups after 14 days of storage at 4 ◦C. *p < 0.05; **p < 0.01; ns, not significant (n = 3).

The Encapsulation efficiency (EE) and loading capacity (LC) of Z–L–TFSG NPs (S1-S7) are also shown in Table 1. The EE of TFSG loaded onto zein nanoparticles was only 72.08 % in the absence of lecithin. However, with the addition of lecithin, the EE and LC of the complex NPs gradually increased to 76.24 % and 3.02 %, respectively. The EE of Z–L–TFSG NPs obviously increased from 76.24 % to 95.12 % at the zein:lecithin mass ratio of 1:1, and the corresponding LC was 4.72 %. When the mass ratio of zein:lecithin changed from 1:1 to 1:2, the EE of the NPs changed from 95.12 % to 98.00 %, and the LC changed from 4.72 % to 3.97 %. Our results suggested that the EE and LC can be efficiently improved with the addition of lecithin to zein. Lecithin could help entrap the TFSG adhered to the surface of zein in the complex NPs (Fig. 1). Furthermore, lecithin might form a complex with the free TFSG, which could increase the EE and LC (Sebaaly, Jraij, Fessi, Charcosset, & Greige-Gerges, 2015). A similar finding was reported in a previous study, where lecithin was added to the zein–EGCG nanoparticles, and the EE and LC of EGCG were significantly increased (Xie et al., 2021). Moreover, the main bioactive compound astilbin and its stereoisomers (isoastilbin, neoisoastilbin, and neoastilbin) were also detected; the chromatograms, EE, and LC of astilbin and its stereoisomers were shown in Fig. 2A. The EE and LC of astilbin and its stereoisomers changed in the same manner as TFSG, which suggested that the Z–L system could also increase the encapsulation and loading efficiency of the main bioactive compound in TFSG.

### Storage stability

The change in PDI and particle size was observed to assess the storage stability of the Z–L–TFSG NPs. The change in PDI and particle size and the precipitation of the complex NPs in each group after 14 days of storage are shown in Fig. 2. As shown in Fig. 2D, the PDI and particle size of S1, S2, S3, and S4 significantly changed after 14 days of storage; a large number of sediments were produced in these groups. In groups S5 and S6, with higher concentrations of lecithin, the particle size and PDI changed gradually after 14 days of storage and only a small amount of sediment was produced. The minor change in the particle size suggested that the storage stability of the S5 and S6 complex NPs was good. The PDI values of S5 and S6 were < 0.3, which suggested that the size distribution of the two samples was uniform during storage.

Taken together, 2:3 was selected as the optimal zein: lecithin mass ratio for the Z–L–TFSG NPs (sample S5), which has been associated with a relatively better EE, LC, PDI, particle size, zeta potential, and storage stability than the other samples. Finally, the Z–L–TFSG NPs with a zein: lecithin: TFSG mass ratio of 10:15:1 was used to encapsulate TFSG in the following studies.

### FTIR spectroscopy

**Fig. 3:**
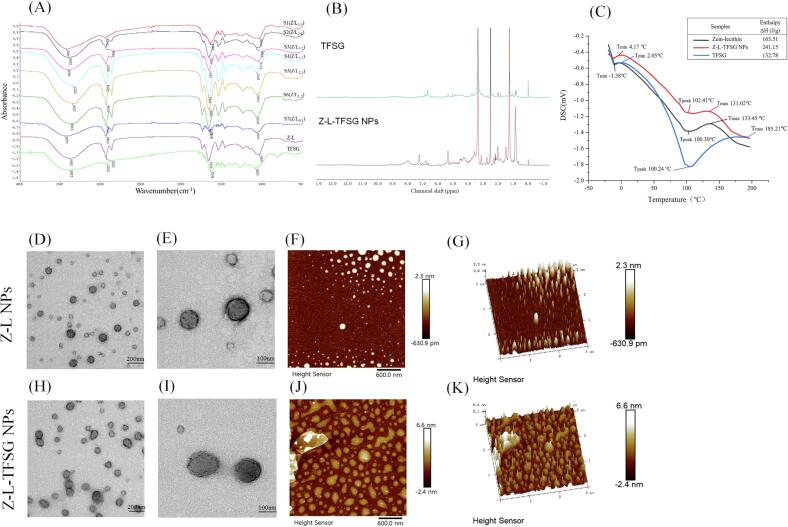
FTIR, NMR, DSC, TEM and AFM of the samples. FTIR (A), ^1^H NMR (B) and DSC (C) spectra of samples. TEM (D, E, H, I), AFM (F, J), and 3D view of AFM (G, K) images of Z-L NPs and Z-L-TFSG NPs, respectively.

The potential intermolecular interactions of TFSG, zein, and lecithin were analyzed by FTIR spectra. According to Fig. 3A, The FTIR spectra of Z–L showed a strong absorption peak at 3360 cm^−1^, which may be attributed to the stretching of the –OH bonds (Miguel A. Cerqueira, 2011).With the addition of TFSG and lecithin, the absorption peak of –OH bonds shifted from 3327 to 3425 cm^−1^, suggesting that hydrogen bonds may have been formed among TFSG, zein, and lecithin. The characteristic peaks of flavones did not appear after their interactions with the protein, which suggested that TFSG might have combined with zein and lecithin by hydrophobic effects or hydrogen bonding. Generally, the characteristic peaks of the protein occurred between 1600 and 1690 cm^−1^ (amide I band) and 1480–1575 cm^−1^ (amide II band). The amide I and II bands of the TFSG were 1661 and 1089 cm^−1^, respectively. The vibration spectra of the amide I band of Z–L–TFSG NPs in different groups were observed to be shifted from 1661 to 1658 cm^−1^ and the amide II band from 1044 to 1089 cm^−1^. These variations may be because of the hydrophobic and electrostatic interactions between zein–lecithin or zein with TFSG (Miguel, Bartolomenu, José, & António, 2011). Above all, zein, lecithin, and TFSG could form stable Z–L–TFSG NPs via hydrophobic, hydrogen bonding, and electrostatic interactions.

### NMR measurement

NMR spectroscopy is one of the primary methods to investigate the hydrogen bonding interactions in solution. The sensitivity of ^1^H chemical shifts and peak shape changed in the electronic environment makes it a useful probe for detecting hydrogen bonded protons. In order to investigate the existence of intermolecular hydrogen bonds, we performed ^1^H NMR spectroscopy study both TFSG and Z–L–TFSG NPs in deuterated DMSO (Fig. 3B).

It was well known that astilbin, isoastilbin, neoisoastilbin, neoastilbin were the main flavonoid compounds in TFSG. The ^1^H NMR spectra of TFSG showed all characteristic peaks of flavonoids after encapsulated with lecithin-zein, and discernible changes was also observed in the aromatic signals δ ∼ 7.3–6.4 (Ar–H) and in the shape and linewidth of the phenolic proton. Generally, the ^1^H NMR resonances of phenol –OH groups in flavonoid compounds could be observed as a significant proton peak signal in aprotic solvents, e.g. OH (5) group, which was the most deshielded signal at *δ* 12.15 ppm due to its participation in a strong intramolecular hydrogen bond of the C (5) OH• • •OC (4) moiety in the A ring, the same was also observed at C (3́) OH and C (4́) OH (*δ* 8.6 ppm). However, the ^1^H NMR resonances of –OH groups would appear as broad signals especially in protic solvents, due to fast exchange of the –OH protons with the solvent, or with the hydrogen bond between different molecules in the aprotic solvents. In our study, the ^1^H NMR resonances of phenol –OH groups display broad signals after encapsulated the TFSG with lecithin-zein in deuterated DMSO–d6, which may be attributed to the intermolecular exchange of phenol –OH proton from flavonoid with various –OH groups protons of the lecithin-zein. The significant proton peak signal change of 5́–OH and 3́–OH also suggest it formed a stable hydrogen bond between TFSG and lecithin-zein. Meanwhile, the aromatic proton signals of TFSG shift downfield from *δ* 7.0 ppm to *δ* 7.2 ppm and from *δ* 6.7 ppm to *δ* 6.8 ppm for H-2′ and H-5′, H-6′ from free to bound state, respectively. The minor changes support indicated aromatic protons of TFSG may be exposed in the hydrophobic cavity of zein.

### DSC analysis

The DSC results of pure TFSG, Z–L, and Z–L–TFSG NPs are shown in Fig. 3C.

The DSC curve of TFSG showed a strong endothermic peak at around 100.24 °C (T_max_ 185.21 °C, T_min_ 2.05 °C), which may be attributed to its melting point and indicated the loss of its crystalline structure (Sebaaly et al., 2015). The characteristic endothermal peak of TFSG was not detected in Z–L–TFSG NPs as shown in Fig. 3C, indicating that TFSG in the amorphous form dissolved in the NPs.

As shown in Fig. 3C, Z–L had a broad absorption peak at around 100.30 °C (T_max_ 133.45 °C, T_min_ −1.38 °C); however, the peak of the thermogram became broader for Z–L–TFSG NPs. The broader peaks indicated higher heterogeneity (range of molecular weights melting) in the sample, which, in turn, confirmed encapsulated TFSG in the Z–L dispersions. As may be seen in Fig. 3C, the denaturation temperature of Z–L–TFSG NPs was higher than that of TFSG and Z–L. The observation may be attributed to the fact that the addition of lecithin increases the electrostatic interaction and hydrophobic effects of the components in the NPs, which, in turn, lead to the high endothermic peak temperature and improve the thermal stability of the complex nanoparticles. Moreover, the enthalpy change (ΔH) of TFSG, Z–L dispersions, and Z–L–TFSG NPs were 132.78, 163.51, and 241.15 J/g, respectively (Fig. 3C). The ΔH is affected by the presence of different components including hydrophobic material, and the changes in ΔH implied that there are chemical or physical interactions in the NPs mixtures. Therefore, the increased ΔH in Z–L–TFSG NPs may due to the extra bonds formed between the Z–L dispersions and Z–L molecules.

### Microstructures by TEM and AFM

The microstructures of freshly fabricated Z–L NPs and Z–L–TFSG NPs were observed by TEM and AFM (Fig. 3). As seen in the TEM images, the Z–L NPs were found to be spherical with a smooth surface and diameters of around 100 nm (Fig. 3D and E); the observation was consistent with the results of DLS. The Z–L–TFSG NPs showed a spherical shape with uniform diameters of around 130 nm (Fig. 3H and I), which might be because of attachment onto the surface of zein NPs. Moreover, the determination of zeta potential confirmed that anionic lecithin might have been deposited on the surface of zein NPs due to the electrostatic attractions.

The morphological structure presented by AFM in Fig. 3 (F, G, J, K) also confirmed the TEM results. The AFM images revealed the Z–L NPs to be spherical with uniform size, with the sample of Z–L–TFSG NPs showing a larger size. We found that some of the Z–L–TFSG NPs had geometrically irregular shapes, which may be attributed to the Z–L–TFSG NPs being clumped and connected. A similar result was also reported by previous studies (Miguel, Bartolomenu, José, & António, 2011). The root-mean-square surface roughness (R_ms_) was used to evaluate the surface roughness of the samples. As shown in Fig. 3F and G, the surface of the Z–L NPs was relatively smooth (R_ms_ = 2.3 nm). After loading with TFSG, the R_ms_ of the Z–L–TFSG NPs increased to 6.6 nm, which was 2.2 times that of the thickness of Z–L NPs. The morphological observation indicated that the Z–L–TFSG NPs were prepared and well distributed.

### Controlled release of TFSG

**Fig. 4:**
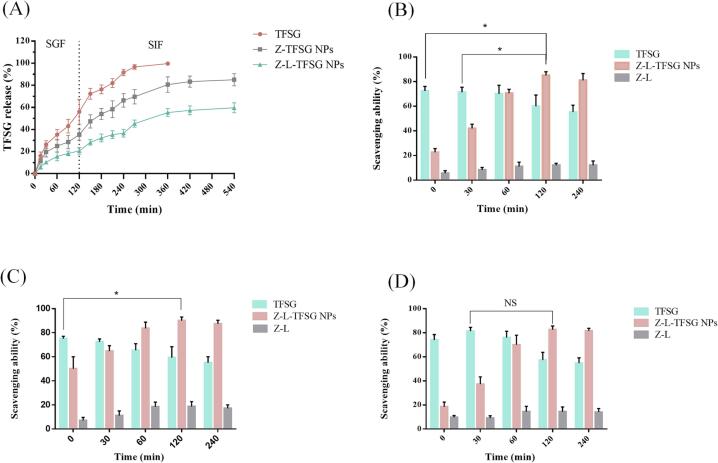
The release rate and antioxidant activity of the NPs. The release rate of the NPs in simulated gastrointestinal environment (A). DPPH (B), ABTS (C), and ·OH (D) scavenging activity. *p < 0.05; NS, not significant (n = 3).

The complex NPs were subjected to a simulated gastrointestinal condition to evaluate the release behavior of TFSG from Z–L–TFSG NPs (Fig. 4A). The cumulative release of TFSG from Z–L–TFSG NPs in SGF gradually increased with the increase in the digestion time and reached approximately 20.6 % after 2 h. However, the cumulative release of TFSG from Z-TFSG NPs was about 35.3 % after 2 h of digestion. In SIF, with increasing digestion time, the cumulative release of TFSG from Z–L–TFSG NPs was also lower than that of Z–TFSG NPs. This was probably owing to the electrostatic interaction between zein and lecithin in the acidic environment (Ahmad et al., 2018, Caporaletti et al., 2017). 56.0 % of free TFSG was released quickly from Z–L–TFSG NPs in SGF after being digested for 2 h, and the residual 44.0 % was also quickly released after digestion in SIF for 6 h. The release rate of encapsulated TFSG significantly decreased in the present study, which suggested that the encapsulation exhibited a controlled release effect. The release rate of TFSG in Z–L complex nanoparticles was the lowest among TFSG, Z–TFSG NPs, and Z–L–TFSG NPs. Therefore, it may be concluded that Z–L–TFSG NPs provided better protection to encapsulated TFSG, which may improve the stability of TFSG in the gastrointestinal tract.

### In vitro antioxidant activity

The *in vitro* antioxidant activity of TFSG, Z–L–TFSG NPs, and Z–L is shown in Fig. 4. In the beginning, the scavenging capacity of TFSG to DPPH⋅ and ⋅OH radicals was the highest, 72.0 % and 75.0 %, respectively. With the increase in the reaction time, the antioxidant capacity of TFSG on ⋅ DPPH⋅ and ⋅OH radicals decreased owing to their instability and easy degradation in the reaction environment. On the contrary, the scavenging capacity of Z–L–TFSG NPs on DPPH⋅ and ⋅OH radicals was the lowest at the beginning of the reaction, after which it increased. After incubation for 120 min, the antioxidant activities of the Z–L–TFSG NPs to DPPH⋅ and ⋅OH radicals were found to be the highest, which was significantly higher than that of free TFSG (*P* < 0.05). Z–L–TFSG NPs and TFSG also exhibited a similar scavenging ability during ABTS measurement, but the difference in the greatest scavenging ability between Z–L–TFSG NPs and TFSG was not significant. Our result implied that TFSG was unstable in the reaction environment and that the Z–L layers had a protective effect on TFSG. Moreover, our study suggested that the TFSG encapsulated by Z–L NPs could enhance its antioxidant activity. This could be due to the aromatic amino acids such as phenylalanine, tyrosine, and tryptophan in zein; the role of these aromatic amino acids was demonstrated using the heat-induced technique. Thus, these aromatic amino acids could increase the antioxidant capacity of these proteins (Song et al., 2022). Our results suggest that the encapsulation of TFSG in Z–L–TFSG NPs could be an efficient method to enhance its antioxidant ability.

### In vitro cytotoxicity

**Fig. 5:**
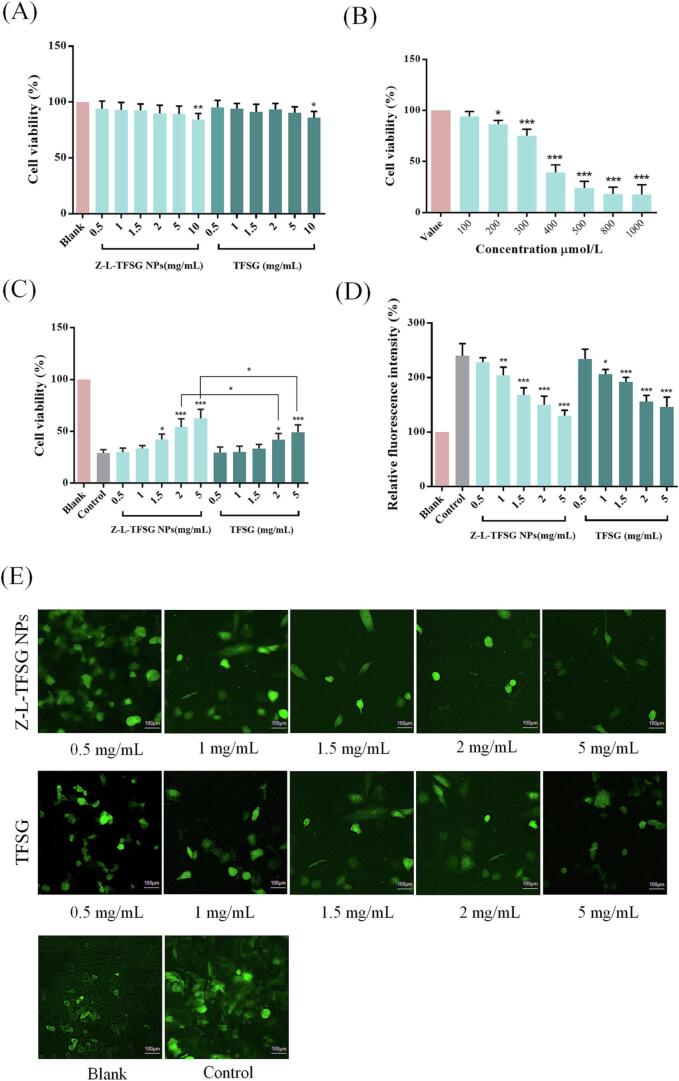
Effect of different concentrations of TFSG and Z-L-TFSG NPs on the viability of HepG2 cells (A); effect of different concentrations of H_2_O_2_ on the viability of HepG2 cells (B); effect of different concentrations of TFSG and Z-L-TFSG NPs on oxidative damage of HepG2 cells induced by H_2_O_2_ (C); Fluorescence staining analysis of ROS production (E) and the corresponding fluorescence intensity (D) in HepG2 cells under fluorescent microscope using a fluorescent microplate reader. *p < 0.05, **p < 0.01, ***p < 0.001 vs control group (n = 5).

The CCK-8 assay was applied to evaluate the potential cellular cytotoxicity of Z–L–TFSG NPs at different concentrations. As presented in Fig. 5A, TFSG and Z–L–TFSG NPs showed similar effects on cell activity in the range of 0.5–10 mmol/L. Among the tested concentrations, TFSG and Z–L–TFSG NPs did not show obvious cytotoxicity in the range of 0.5–5 mmol/L. Over 89 % of the cells treated with ≤10 mmol/L Z–L–TFSG NPs survived after incubation for 24 h, which can be considered in the subsequent experiments as non-toxic concentrations. Significant cytotoxicity was observed after exposure to TFSG or Z–L–TFSG NPs at a concentration of 10 mmol/L for 24 h. The cell survival rates reduced to 84–86 %, showing only slight cytotoxicity against the HepG2 cells. Our results suggested that Z–L–TFSG NPs could not increase the growth inhibition of TFSG on HepG2 cells, which were considered to be relatively safe.

### Protective effects of Z–L–TFSG NPs against H_2_O_2_-induced oxidative damage

As a kind of reactive oxygen species (ROS), H_2_O_2_ can enhance oxidative stress, induce lipid peroxidation, and lead to cell damage or ultimately death·H_2_O_2_-induced oxidative damage has been widely used to evaluate the therapeutic effects of many bioactive substances on oxidative stress. HepG2 cells are usually selected to establish antioxidant models to evaluate the antioxidant capacity of bioactive extraction. Therefore, we studied the protective effects of TFSG and Z–L–TFSG NPs on the H_2_O_2_-induced oxidative damage of HepG2 cells (He, Bu, Xie, & Liang, 2019). As illustrated in Fig. 5B, in the range of 100–1000 μmol/L, HepG2 cell viability decreased with increasing H_2_O_2_ concentration, exhibiting dose-dependent toxicity. When the concentration of H_2_O_2_ was 400 μmol/L, the cell viability of the HepG2 cells decreased to 39.2 %, which indicated that the oxidative damage induced by H_2_O_2_ was serious Thus, 400 μmol/L was chosen as the experimental concentration to establish oxidatively damaged models.

Fig. 5C shows that TFSG and Z–L–TFSG NPs could inhibit the H_2_O_2_-induced oxidative stress. In the control group (H_2_O_2_-injured group) treated with only H_2_O_2_, the cell viability was 29.2 % of that of the blank group. With the increase in the TFSG and Z–L–TFSG NP concentration in the experimental group, both the protective effect for oxidative damage and the ability to inhibit oxidative stress on the cells were improved, suggesting that the protective effects of both TFSG and Z–L–TFSG NPs on cell viability were concentration-dependent. When the concentration of Z–L–TFSG NPs was 2 and 5 mg/mL, the cell viability enhanced to 54.2 % and 62.6 % respectively, which was notably higher than that of TFSG. Our results suggested that TFSG encapsulated by the Z–L NPs could enhance the protective effects of TFSG against H_2_O_2_-induced oxidative damage. Together with the cytotoxicity study, our results confirmed that the protective effects of TFSG and Z–L–TFSG NPs on HepG2 cells were achieved by inhibiting oxidative stress rather than by stimulating the proliferation of HepG2 cells.

### Inhibitory action of the H_2_O_2_-induced damage by ROS on HepG2 cells

2,7-Dichlorodi-hydrofluorescein diacetate (DCFH-DA) is a kind of fluorescent dye without any fluorescence itself, which can penetrate the cell membrane freely. The level of ROS in HepG2 cells can be evaluated by determining the fluorescence intensity of 2′,7′-dichlorofluorescein (DCF) (He et al., 2019). The stronger the fluorescence intensity in fluorescence pictures, the higher the ROS level in the cells. In the present study, the DCFH-DA fluorescence probe was applied to detect the ROS level in the HepG2 cells. After treatment with H_2_O_2_, the fluorescence intensity of DCF increased significantly, which indicated that the H_2_O_2_-induced ROS emerged in the HepG2 cells. In the control group (H_2_O_2_-injured group), H_2_O_2_ induced a significant increase of ROS in the HepG2 cells, which led to a 2.4-fold enhancement in the fluorescence intensity compared with the blank group. In the experimental group, TFSG and Z–L–TFSG NPs had similar effects on ROS, and the fluorescence intensity decreased with increasing TFSG or Z–L–TFSG NPs doses, exhibiting a dose-dependent manner (Fig. 5D and E). When the dose of TFSG and Z–L–TFSG NPs was >1 mg/mL, the fluorescence intensity was obviously lower than that of the control group. Our results suggested that TFSG and Z–L–TFSG NPs could inhibit the damage caused by ROS to HepG2 cells. Z–L–TFSG NPs tend to be associated with an inhibition effect higher than that of TFSG, but the difference was not significant.

### Inference of the formation mechanism of Z–L–TFSG NPs

Herein, we proposed a schematic diagram to elucidate the possible formation mechanism of Z–L–TFSG NPs in Fig. 1. It was reported that zein could self-assemble into colloidal particles when zein and curcumin in aqueous ethanol solutions were dropped into deionized water. With the increasing addition of lecithin, the interaction among zein, lecithin and curcumin was increased (Dai et al., 2017). In our study, based on the results of zeta-potential, turbidity measurement, DSC, FTIR, as well as the morphological observation by TEM and AFM, we proposed the hypothesis of the formation mechanism of Z–L–TFSG NPs. As shown in Fig. 1, the alkyl chain of lecithin was interacted with zein and TFSG mainly through electrostatic interaction, hydrogen bonding, and hydrophobic effects, and formed a more compact structure, which may result in the smaller size of the nanoparticles. It also enhanced the storage stability and increased the LE and EE of TFSG.

## Conclusions

In the present study, TFSG was extracted and purified. Z–L–TFSG NPs were prepared using the ASCP technique. Under the optimal mass ratio of zein:lecithin:TFSG (10:15:1), the Z–L–TFSG NPs had a particle size of approximately 131 nm, a PDI of approximately 0.18, a zeta potential of approximately –44.1 mV, and an EE of 98.0 %. The nanoparticles were characterized by DSC, FTIR, TEM, and AFM. Z–L–TFSG NPs showed superior stability and better controlled release property in simulated gastrointestinal digestion. The encapsulation of TFSG in Z–L NPs could improve its *in vitro* antioxidant capacity. Moreover, Z–L–TFSG NPs could enhance the protective effects of TFSG against H_2_O_2_-induced oxidative damage to HepG2 cells. Based on these results, it may be concluded that the encapsulation of TFSG in Z–L NPs could improve the stability and show a more significant effect on the antioxidant capacity than that of TFSG. As such, the Z–L self-assembled NPs could be developed as a promising delivery system for the integrated encapsulation of multiple flavonoids in food and drugs.

## Funding

This project was funded by grants from the National Natural Science Foundation of China [NO. 81960705], the National Key Research and development Program of China [NO. 2019YFC1712503], Southwest Minzu University Basic Scientific Research Business Special Fund project for Central Universities [NO. ZYN2023006].

## CRediT authorship contribution statement

**Jing Li:** Methodology, Writing – original draft. **Yingxiu Zhang:** Formal analysis, Methodology. **Wenfang Jin:** Formal analysis, Methodology. **Yue Wang:** Methodology, Data curation. **Li Yang:** Methodology, Data curation. **Zhifeng Zhang:** Conceptualization, Supervision, Writing – review & editing. **Zhigang Yan:** Investigation, Writing – review & editing.

## Declaration of Competing Interest

The authors declare that they have no known competing financial interests or personal relationships that could have appeared to influence the work reported in this paper.

## Data Availability

Data will be made available on request.
